# Epidemiological characteristics and risk factors of suspected and confirmed mpox cases during the 2022–2023 epidemic in the Capital Region, Korea

**DOI:** 10.4178/epih.e2024092

**Published:** 2024-11-24

**Authors:** Mingyeol Shim, Soo Hyeon Cho, Seung Eun Lee, Taeyoung Kim

**Affiliations:** 1 Korea Disease Control and Prevention Agency, Cheongju, Korea; 2 Division of Infectious Disease Response, Capital Regional Center for Disease Control and Prevention, Korea Disease Control and Prevention Agency, Seoul, Korea; 3 Department of Nursing, Yonsei University, Seoul, Korea

**Keywords:** Mpox (monkeypox), Sexually transmitted diseases, Human immunodeficiency virus

## Abstract

**OBJECTIVES:**

This study investigated the general characteristics of laboratory-confirmed mpox patients in the Capital Region of Korea, as well as the risk factors for mpox infection, particularly focusing on the characteristics of polymerase chain reaction (PCR)-positive and PCR-negative cases.

**METHODS:**

We investigated 160 adults, excluding 4 minors, from 164 suspected mpox patients reported in Seoul, Gyeonggi, Incheon, and Gangwon from June 21, 2022 to October 31, 2023. Data were collected via telephone and face-to-face interviews. A statistical analysis of the general characteristics of the infection was conducted using frequency analysis and logistic regression.

**RESULTS:**

Of the 160 suspected cases of mpox, 59.3% (n=95) tested positive via mpox-PCR. Among the confirmed cases, 97.9% (n=93) were male. PCR-positive patients typically presented with genital and anal skin rashes or mucosal lesions, accompanied by pain. Additionally, 35.5% (n=33) of the male patients had human immunodeficiency virus (HIV) infections. Most confirmed cases (94.7%, 90/95) were believed to have contracted mpox through sexual contact during the maximal incubation period of 21 days prior to symptom onset, with a significant number reporting same-sex or casual contact. The most commonly collected and highest-yielding specimens from PCR-positive patients were from skin or mucosal lesions, whereas blood samples demonstrated the lowest percent positivity.

**CONCLUSIONS:**

In the Capital Region, most PCR-positive cases were male patients in their 30s who had sexual contacts and exhibited symptoms, aligning with findings from previous studies. These results provide a foundation for the differential diagnosis concerning mpox infection and the selection of PCR-test samples in clinical settings.

## INTRODUCTION

Mpox (previously known as monkeypox virus infection) is a zoonotic disease that is transmitted from animals to humans. Traditionally, it has been endemic to the tropical rainforest regions of West and Central Africa, where the virus-carrying animals reside [1,2]. However, since May 2022, mpox has been detected in non-endemic regions, including Europe and North America, where transmission through animal contact is not commonly reported [3,4]. In contrast to earlier outbreaks, a significant majority (85.5%) of recent cases have occurred among men who have sex with men [5]. According to the latest data from the World Health Organization as of April 2024, there have been 97,208 cumulative confirmed cases and 186 deaths globally [5]. In Korea, the first case was identified on June 21, 2022 and by December 31, 2023, there have been 155 reported cases with no fatalities [6]. While there have been isolated reports of healthcare-associated mpox infections [7,8] and studies examining the clinical characteristics of patients [9], no research has yet been conducted to assess the risk factors for mpox infection based on the suspected cases reported in Korea.

The purpose of our study was to identify the epidemiological, diagnostic, and clinical characteristics, as well as differences between positive and negative cases, focusing on suspected cases in the Capital Region (Seoul, Gyeonggi, Incheon, Gangwon). Through this analysis, the study aims to understand the characteristics of mpox patients and suggest enhancements to the national surveillance system.

## MATERIALS AND METHODS

### Study subjects

The study analyzed 160 suspected mpox cases reported from June 21, 2022 to October 31, 2023, in the Capital Region area, which includes Seoul, Gyeonggi, Incheon, and Gangwon. Each individual was classified as a suspected case by city and provincial epidemiological investigation officers and underwent mpox–polymerase chain reaction (PCR) testing to confirm the diagnosis. When test results were positive, the patients were treated and isolated.

### Data collection

Data were collected through telephone or face-to-face interviews with 160 suspected cases by city and provincial epidemiological investigation officers in accordance with the national mpox response guidelines [10]. The responses from these interviews, along with medical records, were reviewed to identify each patient’s gender, age, risk factors (such as recent travels, timing, location, and mode of suspected exposures), date of symptom onset, and medical history, including human immunodeficiency virus (HIV) infection status.

### Case definition

According to the mpox response guidelines issued by the Korea Disease Control and Prevention Agency (KDCA) [10], a suspected patient is defined as someone who exhibits clinical symptoms and an epidemiological link suggesting mpox infection but whose diagnostic test results do not meet the criteria. This category also includes individuals without a confirmed epidemiological link to mpox, such as overseas travel or sexual contact history, but who display clinical symptoms typical of mpox, including skin rashes on the anorectum, genitalia, oral cavity, conjunctiva, or urethra, as well as anal or genital pain. Additionally, it encompasses individuals with a strong epidemiological link to mpox, such as sexual contact with a symptomatic person, but who only exhibit non-specific symptoms like chills, myalgia, sore throat, fever, sweating, fatigue, headache, body aches, back pain, and lymphadenopathy. Clinical manifestations include an acute rash or pain of unknown origin on the skin or mucous membranes, accompanied by 1 or more of the following symptoms: acute fever (temperature ≥38.5°C), headache, lymphadenopathy (inflammation or enlargement), back pain, myalgia, or dysuria [10]. Epidemiological linkage is assessed based on criteria such as contact with a patient with confirmed or suspected mpox within 21 days prior to symptom onset, travel to a country with an mpox epidemic or an area where cases have been reported since May 2022, or sexual contact with multiple or casual partners. Real-time PCR testing [11] is conducted according to diagnostic protocols, with samples collected from skin lesions, body fluids and tissues, crusts, blood, or oropharyngeal swabs, and tested by a local public health and environmental laboratory. A confirmed case is defined as an individual with clinical symptoms consistent with mpox and a positive result on a diagnostic test.

### Statistical analysis

Based on the collected data, frequency analyses were conducted to examine the characteristics of mpox cases and diagnostic test samples. Binomial logistic regressions were used to compare PCR-positive and PCR-negative cases and to estimate odds ratios (ORs). Statistical analyses were performed using R version 4.1.2 (R Foundation for Statistical Computing, Vienna, Austria).

### Ethics statement

The study received approval from the Institutional Review Board of the KDCA (IRB No. KDCA-2023-10-03-P-01).

## RESULTS

### Mpox case reports in Korea

Since the initial report of mpox nationwide in June 2022, there have been 117 confirmed cases by October 31, 2023. Of these, 95 cases occurred in the Capital Region, representing 81.2% (95 out of 117) of the total confirmed cases in Korea. Additionally, during this period, the positive predictive value of suspected case reports in the Capital Region was 59.4% (95 confirmed cases out of 160 suspected cases).

### General characteristics of suspected mpox cases in the Capital Region area

In examining the characteristics of 160 suspected patients in the Capital Region, the majority were male (95.6%, n=153) and resided in Seoul (61.2%, n=98), followed by those living in Gyeonggi, Incheon, and Gangwon. The average age was 34.8 years (standard deviation [SD], 9.7), including 3 cases over the age of 60. When comparing PCR-positive and negative cases, over 90% of individuals in both groups were male (97.9 and 92.3%, respectively), with average ages of 34.1 years (SD, 7.8) and 35.9 years (SD, 11.9), respectively. Among age groups, the largest segment of PCR-positive cases was in their 30s (50.5%, n=48), while the largest segment of PCR-negative cases was in their 20s (46.2%, n=30). Regarding risk exposure history, 136 cases (85.0%, 136 out of 160) reported sexual contacts, and 1 case (0.6%) reported exposure during a medical procedure, with no exposure reported among 23 individuals (14.4%, 23 out of 160). Comparing the PCR-positive and negative groups, 94.7% (90 out of 95) of positive patients had histories of sexual contact, whereas 70.8% (46 out of 65) of negative patients reported the same. Among those who reported sexual contact, same-sex contact constituted the largest proportion at 61.8% (84 out of 136), while opposite-sex contact was reported in 7.3% (n=10). No bisexual contact was reported. The PCR-positive group showed a higher proportion of same-sex contact at 88.9% (80 out of 90), with 2.2% (2 out of 90) refusing to report. Conversely, in the PCR-negative group, same-sex and opposite-sex contacts were reported at 8.7% (4 out of 46) and 4.3% (2 out of 46), respectively, while 87.0% (40 out of 46) refused to report.

The most common symptom observed was a skin rash, which appeared in all PCR-positive cases and in 99.4% of PCR-negative cases. Other symptoms among PCR-positive patients included fever (60.0%), chills (49.8%), and myalgia (49.8%). In PCR-negative cases, the symptoms were fever (40.0%), sore throat (26.7%), and chills (23.1%). On the day symptoms began, 47.4% of PCR-positive patients exhibited systemic symptoms, 29.5% had skin or mucous lesions, and 23.1% had both. In contrast, PCR-negative cases more commonly presented with both types of symptoms on the onset day (46.2%), followed by skin and mucous lesions (29.2%) and systemic symptoms (24.6%). This highlights the differences in symptom presentation between PCR-positive and PCR-negative cases. Both PCR-positive and PCR-negative cases were primarily self-reported (67.4% for PCR-positive and 63.1% for PCR-negative cases), with the next most common reports coming from physicians (31.5 and 35.4%, respectively). There was 1 case (1.1%, 1 out of 95) identified due to prior confirmation by a sexual partner (Table 1). Except for 1 needlestick infection, there were no additional confirmed cases among non-sexual contacts of the confirmed cases, such as family members, health care workers, or roommates. The mean incubation period, calculated from the 76 participants who reported the date of their last sexual contact, was 9.3 days. The incubation period ranged from a minimum of 1 day to a maximum of 49 days.

### Analysis of risk factors of mpox infection

The risk of infection, as indicated by PCR positivity, did not vary significantly among groups categorized by the number of sexual contacts. Individuals who had casual sexual contacts exhibited a higher risk (OR, 10.35; 95% confidence interval [CI], 2.75 to 38.97) compared to those with regular partners. There was no significant difference in risk between cases with same-sex contact (OR, 5.00; 95% CI, 0.79 to 31.70) and those with opposite-sex contact. Furthermore, individuals with HIV infection faced a higher risk (OR, 2.13; 95% CI, 1.02 to 4.46) than those without HIV infection. Cases presenting with lesions in the anal or genital areas were at a higher risk (OR, 7.81; 95% CI, 3.79 to 16.10) compared to those without such lesions. Similarly, cases experiencing pain in the lesions were at higher risk (OR, 5.23; 95% CI, 2.49 to 11.20) than those without pain (Table 2).

### Analysis of percent positivity by sample type

The characteristics of test samples from PCR-positive cases were analyzed, as shown in Table 3. Skin or mucosal lesion samples, which were collected from 98.9% (94 out of 95) of the cases, exhibited the highest percent positivity at 96.8%. Scab samples, collected from 33.7% (32 out of 95) of the cases, demonstrated a percent positivity of 81.3%. Oropharyngeal swab samples, obtained from 83.2% (86 out of 95) of the cases, showed a percent positivity of 64.0%, with 8.1% yielding inconclusive results. Blood samples, also collected from 98.9% of the cases, displayed the lowest percent positivity at 9.3%, with 12.5% of the results being inconclusive.

## DISCUSSION

This study aimed to provide essential information for the development of mpox surveillance and control strategies by analyzing the characteristics of mpox patients reported in the Capital Region. These characteristics encompassed demographics, risk exposures including sexual contact, HIV infection status, and clinical symptoms. The findings revealed that 59.4% (95 out of 160) of the suspected cases tested positive, predominantly among males in their 30s. Consistent with prior research, factors such as same-sex contact, multiple or casual sexual partners, and HIV infection were commonly reported among PCR-positive cases. Notably, in our study, casual contacts and HIV infection emerged as significant risk factors for mpox infection [12–15]. Regarding symptoms, many patients initially experienced systemic symptoms, particularly chills and myalgia. Additionally, in PCR-positive cases, skin rashes and mucosal lesions frequently appeared in the anal and genital areas, aligning with previous studies [16–18] that highlighted the correlation between the location of rashes and mpox infection. Notably, PCR-positive cases reported more frequent pain in the lesions compared to PCR-negative cases, suggesting that the location of lesions and the presence of pain could serve as indicators for suspecting mpox infection. This insight could augment existing research that describes the clinical symptoms of confirmed cases [19,20], offering valuable guidance for clinical judgment in medical practice.

The characteristics of mpox-PCR results were also presented. Skin and mucosal lesion samples were the most frequently collected and exhibited the highest positivity rates among the sample types, indicating that most confirmed cases presented with skin rashes [9]. Additionally, the low frequency of scab sample testing is likely due to several factors: the potential absence of scab formation depending on the patient’s condition, the risk of secondary infection, patient discomfort, and the possibility of scarring during the collection process. Blood samples, although collected as frequently as skin and mucosal lesions, demonstrated the lowest positivity rates. These findings could serve as guidelines for prioritizing types of samples during collection in clinical settings.

This study has several limitations. First, although in-depth epidemiological investigations were conducted for PCR-positive cases, yielding high-quality information, only initial investigations were carried out for PCR-negative cases. This may have resulted in insufficient data. Notably, the high proportion of PCR-negative cases where the type of sexual contact could not be identified suggests a tendency to withhold sensitive information during the initial investigation phase. Consequently, caution is advised when generalizing these findings to all suspected mpox cases. Second, for mpox, certain conditions, such as high-risk groups or specific exposure contexts, may result in cases and controls exhibiting similar characteristics. This similarity can complicate the interpretation of high-risk ratios, making it challenging to pinpoint specific risk factors for disease transmission. Third, data regarding the last sexual contact within 21 days were available for only 76 of the 95 positive cases. When multiple sexual contacts occurred within this period, pinpointing the exact date of exposure to the infection source proved challenging.

This study is significant as it is the first in the country to analyze all reported suspected mpox cases, compare the characteristics of PCR-positive and PCR-negative cases, and assess the associated risk factors for infection. With mpox being reclassified as a third-grade infectious disease under the Infectious Disease Control and Prevention Act effective January 1, 2024, changes in the surveillance system [21] are anticipated, which may lead to a relaxation of disease control measures. This underscores the importance of including mpox in clinical differential diagnoses to ensure thorough surveillance. The findings of this study are expected to provide a robust basis for conducting differential diagnoses of mpox and for implementing mpox-PCR testing with appropriate specimen types.

## Figures and Tables

**Table 1:** General characteristics of suspected patients in the Capital Region

Characteristics	PCR-positive (n=95)	PCR-negative (n=65)	Total (n=160)
Sex			
Male	93 (97.9)	60 (92.3)	153 (95.6)
Female	2 (2.1)	5 (7.7)	7 (4.4)
Area of residence			
Seoul	65 (68.4)	33 (50.8)	98 (61.2)
Gyeonggi	22 (23.2)	25 (38.5)	47 (29.4)
Incheon	6 (6.3)	4 (6.2)	10 (6.3)
Gangwon	2 (2.1)	3 (4.6)	5 (3.1)
Age (yr)			
Mean± SD	34.1±7.8	35.9±11.9	34.8±9.7
20–29	33 (34.7)	30 (46.2)	63 (39.4)
30–39	48 (50.5)	14 (21.5)	62 (38.7)
40–49	9 (9.5)	11 (16.9)	20 (12.5)
50–59	4 (4.2)	8 (12.3)	12 (7.5)
≥60	1 (1.1)	2 (3.1)	3 (1.9)
Risk exposure			
Sexual contact	90 (94.7)	46 (70.8)	136 (85.0)
Healthcare-associated infection	1 (1.1)	0 (0.0)	1 (0.6)
Unknown^1^	4 (4.2)	19 (29.2)	23 (14.4)
Type of sexual contact^2^			
Same-sex	80 (88.9)	4 (8.7)	84 (61.8)
Opposite-sex	8 (8.9)	2 (4.3)	10 (7.3)
Not available	2 (2.2)	40 (87.0)	42 (30.9)
Symptoms^3^			
Fever	57 (60.0)	26 (40.0)	83 (51.9)
Chill	47 (49.8)	15 (23.1)	62 (38.8)
Headache	19 (20.0)	13 (20.0)	32 (20.0)
Fatigue	14 (14.7)	7 (10.8)	21 (13.1)
Myalgia	47 (49.8)	14 (21.5)	61 (38.1)
Lymphadenopathy	18 (18.9)	7 (10.8)	25 (15.6)
Sore throat	17 (17.9)	18 (26.7)	35 (21.9)
Skin rash or mucosal lesions	95 (100)	64 (98.5)	159 (99.4)
Onset symptom			
Systemic symptoms	45 (47.4)	16 (24.6)	61 (38.1)
Skin rash or mucosal lesions	28 (29.5)	19 (29.2)	47 (29.4)
Concurrent^4^	22 (23.1)	30 (46.2)	52 (32.5)
Person who notified			
Self	64 (67.4)	41 (63.1)	105 (65.6)
Sexual partner	1 (1.1)	1 (1.5)	2 (1.3)
Physician	30 (31.5)	23 (35.4)	53 (33.1)

Values are presented as number (%).

PCR, polymerase chain reaction: SD, standard deviation.

1 In cases where clinical symptoms consistent with mpox are present, even if epidemiological connections have not been confirmed.

2 Included only those with identified sexual contacts (n=136).

3 If a single patient showed several types of symptoms, they were counted multiple times.

4 Systemic symptoms and skin or mucosal lesions appeared simultaneously.

**Table 2:** Analysis of risk factors for PCR-positive and negative cases

Factors	PCR-positive (n=95)	PCR-negative (n=65)	OR (95% CI)	p-value
No. of sexual partners				
No contact	5	20	0.14 (0.05, 0.41)	<0.001
1	54	30	1.00 (reference)	
2–3	30	12	1.39 (0.62, 3.11)	0.424
≥4	6	3	1.11 (0.26, 4.77)	0.887
Types of sexaul partners				
Casual partner	88	34	10.35 (2.75, 38.97)	<0.001
Regular partner	2	12	1.00 (reference)	
Type of sexual contact^1^				
Same-sex	80	4	5.00 (0.79, 31.70)	0.090
Opposite-sex	8	2	1.00 (reference)	
Not available	2	40	0.01 (0.00, 0.10)	<0.001
HIV infection^2^				
Confirmed	33	13	2.13 (1.02, 4.46)	0.050
Not confirmed	62	52	1.00 (reference)	
Systemic symptoms				
Yes	67	46	0.99 (0.49, 1.98)	0.970
No	28	19	1.00 (reference)	
Skin rash or mucosal lesion^3^				
Anal/genital	77	23	7.81 (3.79, 16.10)	<0.001
Others	18	41	1.00 (reference)	
Pain in lesions^3^				
Yes	49	11	5.23 (2.49, 11.20)	<0.001
No	46	53	1.00 (reference)	

PCR, polymerase chain reaction; OR, odds ratio; CI, confidence interval; HIV, human immunodeficiency virus.

1 Included only those with identified sexual contacts (n=136).

2 A person confirmed to have HIV based on diagnostic test results.

3 Excluding one individual without skin rash (n=159).

**Table 3:** Analysis of percent positivity by sample type in PCR-positive cases (n=95)

Sample type^1^	Collection	Positive (n)	Negative (n)	Pending	Positivity (%)^2^
Skin rash or mucosal lesion	94 (98.9)	91	3	-	96.8
Scab	32 (33.7)	26	6	-	81.3
Oropharyngeal swab	86 (83.2)	55	24	7 (8.1)	64.0
Blood	94 (98.9)	8	78	8 (12.5)	9.3

Values are presented as number (%).

PCR, polymerase chain reaction.

1 If a patient provided multiple specimens, they were considered duplicates.

2 Proportion of positive detections among collected samples.
